# Satellitome Analysis of *Rhodnius prolixus*, One of the Main Chagas Disease Vector Species

**DOI:** 10.3390/ijms22116052

**Published:** 2021-06-03

**Authors:** Eugenia E. Montiel, Francisco Panzera, Teresa Palomeque, Pedro Lorite, Sebastián Pita

**Affiliations:** 1 Department of Experimental Biology, Genetics, University of Jaén. Paraje las Lagunillas sn., 23071 Jaén, Spain; emontiel@ujaen.es (E.E.M.); tpalome@ujaen.es (T.P.); 2 Evolutionary Genetic Section, Faculty of Science, University of the Republic, Iguá 4225, Montevideo 11400, Uruguay; fcopanzera@gmail.com

**Keywords:** Chagas disease vector, *Rhodnius prolixus*, satellite DNA, satellitome, fluorescent in situ hybridization, satellite DNA expression, genome evolution

## Abstract

The triatomine *Rhodnius prolixus* is the main vector of Chagas disease in countries such as Colombia and Venezuela, and the first kissing bug whose genome has been sequenced and assembled. In the repetitive genome fraction (repeatome) of this species, the transposable elements represented 19% of *R. prolixus* genome, being mostly DNA transposon (Class II elements). However, scarce information has been published regarding another important repeated DNA fraction, the satellite DNA (satDNA), or satellitome. Here, we offer, for the first time, extended data about satellite DNA families in the *R. prolixus* genome using bioinformatics pipeline based on low-coverage sequencing data. The satellitome of *R. prolixus* represents 8% of the total genome and it is composed by 39 satDNA families, including four satDNA families that are shared with *Triatoma infestans*, as well as telomeric (TTAGG)_n_ and (GATA)_n_ repeats, also present in the *T. infestans* genome. Only three of them exceed 1% of the genome. Chromosomal hybridization with these satDNA probes showed dispersed signals over the euchromatin of all chromosomes, both in autosomes and sex chromosomes. Moreover, clustering analysis revealed that most abundant satDNA families configured several superclusters, indicating that *R. prolixus* satellitome is complex and that the four most abundant satDNA families are composed by different subfamilies. Additionally, transcription of satDNA families was analyzed in different tissues, showing that 33 out of 39 satDNA families are transcribed in four different patterns of expression across samples.

## 1. Introduction

*Rhodnius prolixus*, due to its medical importance as one of the main Chagas disease vector species, was the first Triatominae species to be sequenced [1]. Assembled sequences covered about 702 Mb, approximately 95% of the genome taking into consideration that the haploid genome size of *R. prolixus* was estimated at 733 Mb [2]. According to the annotation of this genome assembly, the repeatome of *R. prolixus*—repeated DNA sequences composing a genome [3]—make up to 5.6% of the genome, with Class II transposable elements being the main components [1]. Recently, Castro et al. [4] applied dnaPipeTE software [5] to re-evaluate the transposable element (TEs) quantification in the *R. prolixus* genome, with astonishing results. Using the same raw data obtained in the genome assembly project, Castro et al. [4] estimated that the amount of TEs in the *R. prolixus* genome ranged between 19% and 23%, that is, three to four times higher than the original quantification of Mesquita et al. [1]. In addition, they evaluated other sibling species, *R. montenegrensis* and *R. marabaensis* (formerly *R. robustus* II and III, respectively [6]), with similar results. These findings also confirmed that Class II elements were the most abundant TEs in *Rhodnius* genomes [1,7]. The main issue of genomes with large repeatome is that repeated DNA sequences hinder the genome assembly process, resulting in collapsed and fragmented genomes and an underestimation of their amount in the genome [8,9]. This underestimation may be caused by several reasons. First, not all repeated DNAs might be present in the genome assembly, or those present could be collapsed. Second, the methodology used was based solely on homology to already known sequences, which makes it likely that new or highly divergent families will not be detected.

Another important component of the repeatome is tandem repeat DNA, in particular, satellite DNA (satDNA). SatDNA is defined as a non-genic repeat sequence organized in arrays of variable length and it can be classified by its repeat unit length as microsatellites, minisatellites and satellites [10]. Currently, data on satDNA repeats are almost completely missing for the *R. prolixus* genome. The only available information on *R. prolixus* satDNA is the existence of four satDNA families that are shared with other triatomine species, *Triatoma infestans* [11], another important Chagas disease vector. However, there is a wealth of information about satDNAs of *T. infestans*. Firstly, in this species, three AT-rich satDNA families strongly related to transposable elements have been characterized from a C0t library [12]. A few years later, Pita et al. [13] described the *T. infestans* repeatome finding that satDNAs are the main component of the repeated fraction. The *T. infestans* satellitome, the collection of satDNA families in a genome [14], includes 42 different satDNA families [13]. Moreover, satellitome analysis could determine that genome size differences between *T. infestans* major lineages (Andean and non-Andean) were due to variations in satDNA abundance [13]. Recently, the satellitome of another heteropteran, *Holhymenia histrio*, has been described, with 34 satDNA families with great variability in their chromosomal location [15].

Currently, satDNA characterization has experienced a huge increase due to the emergence of bioinformatics pipelines using low-coverage sequencing data, i.e., RepeatExplorer or TAREAN [16,17]. In the last five years, more than 40 studies have been published describing satDNA families using those pipelines, among them, the satellitome analysis of *T. infestans* and *H. histrio*. Other successful examples of the employment of this methodology were the satellitome analyses of several grasshoppers [14,18], one cricket species [19], *Drosophila* [20] or beetles [21]. Outside the arthropods, satDNA characterization from low-coverage sequencing data have been published in fish [22,23,24] and mostly in plants [25,26,27,28], among several other species. Those studies have paved the way for understanding satDNA organization and analyzing new roles in the genomes.

Besides their well-known structural function composing heterochromatin, centromeres or telomeres, a relevant role in chromosomal organization, pairing and segregation has been attributed to satDNA [8,29]. For instance, Cabral-de-Mello et al. [30] have recently reported its involvement in the differentiation of the Z sex chromosome in Crambidae moths. Furthermore, non-coding transcripts of satDNA are involved not only in heterochromatin maintenance and kinetochore assembly [31,32], but also in mosquito embryonic development, promoting gene silencing [33]. In *Drosophila melanogaster*, transcripts derived of the (AAGAG)_n_ satellite are important for viability and male fertility [34], whereas in *Tribolium castaneum*, TCAST satDNA expression affects the epigenetic state of constitutive heterochromatin in heat-shock conditions [35]. In addition, satDNA is transcribed in many insect species during development and exhibits differential expression between tissues or sexes [19,36,37]. Finally, Shatskikh et al. [38] reviewed the transcription of satDNA in *Drosophila*. According to the authors, the generated small RNAs have an important role, among others, in the viability and fertility of the fly and in the regulation of gene expression, also contributing to facilitate dosage compensation.

Herein, we present the description of *R. prolixus* satellitome covering the characterization of their sequences, abundance, divergence and transcriptional activity of each satDNA family. This study is the first to address a genome-wide analysis of the satDNA in this species, contributing to the knowledge of sequences that compose the genome of this important vector of Chagas disease.

## 2. Results and Discussion

The kissing bug *R. prolixus* is the main vector of Chagas diseases in countries such as Colombia and Venezuela in Latin America [39], and the first Triatomine species whose genome has been sequenced and assembled [1]. Here, we described its satellitome, which is composed by 39 satDNA families, 34 of which were detected by RepeatExplorer2 analysis, and 5 were detected by RepeatMasker mapping. Those results will be discussed below.

Low-coverage sequencing was performed obtaining 6,380,542 paired-end reads (932 Mb) after quality trimming processing. RepeatExplorer2 was used for de novo discovery of satDNAs, together with RepeatMasker to estimate their abundance and divergence. A subset of six million reads were used as input for the RepeatExplorer2 run, and six hundred thousand reads (≈90 Mb) were randomly taken as a sample by the software to perform the clusterization (0.12X coverage). Overall, annotation determined that 24% of the genome were multi-copy DNA sequences (Supplementary Figure S1). The analysis of 437 clusters above 0.001% of the genome rendered a total of 34 satDNA families, pointing out that these kinds of tandem repeats bear a great variability.

As commented in the Introduction, only four low copy-number satDNA families have been characterized up to now in the *R. prolixus* genome, all of them shared with *T. infestans* [11]. Although three of these four satDNA families were not detected by the RepeatExplorer2 approach, RepeatMasker masking allowed to confirm their presence. One possible explanation of non-detection of these three satDNA families could be their abundance. Their amount in the *R. prolixus* genome is lower than in the *T. infestans* genome (RproSat13-293, 0.067% vs. TinfSat04-1000, 2.45–4.26%; RproSat25-84, 0.009% vs. TinfSat12-84, 0.02–0.03%; RproSat37-98, 0.0005% vs. TinfSat15-99, 0.01–0.02%) (Table 1). Only the most abundant of the shared satDNAs was present in one cluster obtained by RepeatExplorer2. This satDNA family, RproSat06-136, is one-fold more abundant in *R. prolixus* (0.32%) than in *T. infestans* (TinfSat33-372, 0.01–0.02%). Nevertheless, the amount of two of these families (RproSat13-293 and RproSat25-84) exceeds 0.001% of the genome, which was the limit used for the RepeatExplorer2 analysis, and they should have been detected on the analyzed clusters. It is possible that the high nucleotide diversity observed for these two satDNA families (28.28% and 25.75%) may interfere with the reads’ clustering. No new shared satDNA families have been detected between the two species other than the four previously described. The existence of only four low copy-number satDNA families of *R. prolixus* shared with *T. infestans* is in accordance with genomic in situ hybridization analyses in *R. prolixus* using *Triatoma* genomic probes, which revealed that repetitive DNA between both genera were not shared at a great scale [40,41]. These results reinforce the idea of the great genomic difference between both Triatominae genera. Moreover, this scenario is expected for the fast-evolving satDNA sequences. The library evolution hypothesis states that close species tend to share a group of satDNA families, the so-called “library”, which in turn, during the divergence of species, may change due to the loss or amplification of each member of the library. Hence, the more distant two species are, the less probable it would be that they share satDNA families between them [42,43]. Since Triatomini and Rhodniini divergence was dated around 18–22 million years ago (Mya) [44], it represents a rather high divergence time under the library evolution hypothesis. Other insects showing similar satDNA families presented shorter divergence periods below 8 Mya, i.e., three species from the *Drosophila obscura* subgroup [45], *Drosophila virilis* and *D. americana* [20], or in several grasshopper species from the *Schistocerca* genus [46]. Nevertheless, there are also some exceptional cases of shared satDNAs among ant species, with great divergence time (74–80 Mya) [47]. Considering other biological groups besides insects, there are extreme cases of conserved satDNAs, such as the dodeca satellite present in *Drosophila melanogaster*, *Arabidopsis thaliana* and humans [48], or the BIV160 and DTHS_3_ satDNA families conserved in bivalve mollusks for over 500 My [49].

RepeatExplorer2 analysis has limitations to detect low-complexity sequences, such as the telomeric repeats [16], but RepeatMasker analysis confirmed the presence of the insect canonical telomeric repeat (TTAGG)_n_ in *R. prolixus*—previously reported by FISH [50]—though at low amounts (0.003% of the genome). In consequence, other repetitive DNAs with short repeat units may have been omitted in this analysis. Therefore, (GATA)_n_ repeats amount was calculated since this repeat is extremely abundant in the *T. infestans* genome and it seems to be the only repeat DNA shared in the Y chromosomes in *Triatoma* species [41]. In *R. prolixus*, (GATA)_n_ repeats are barely 0.001% of the genome, far away from the 4.5% in *T. Infestans* [13].

Taking into consideration the telomeric and the (GATA)_n_ repeats, at least 39 satDNA families are present in the *R. prolixus* satellitome, representing 8.05% of its genome (Table 1). For the nomenclature of the different satDNA families, the proposal of Ruíz-Ruano et al. [14] has been followed, with the satDNA family name bearing the species name abbreviation (Rpro), a number in decreasing abundance and the length of the repeat sequence (Table 1, Supplementary Table S1). The relationships between satDNA families were analyzed by comparison of the consensus sequences. Most of the satDNA families did not show similarity with the sequences of other families. However, four satDNA families presented regions with high similarity. RproSat07-375 and RproSat09-499 families share 79 bp with an identity of 82%, while RproSat22-980 and Rpro24-675 share 69 bp with an 88% identity (Supplementary Figure S2). The high similarity found between the sequences of those satDNA families may suggest that they are evolutionarily related in spite of their different size.

In *R. prolixus,* the C-banding technique revealed the existence of heterochromatin only on the Y chromosome, that is completely heterochromatic. Whereas in *T. infestans*, in addition to an entirely heterochromatic Y chromosome, there are prominent autosomal C-heterochromatic regions whose number and size varies between Andean and non-Andean lineages [2,11,51,52]. This corroborates the higher proportion of satDNA in *T. infestans* (33% and 25% in Andean and non-Andean genomes, respectively) [13] in relation to *R. prolixus* (8.05%). Regarding *R. prolixus*, the amount of the satDNA families descends gradually, with the top family being 2.13%, and just nine families above 0.1%. On the other hand, *T. infestans* genomes present few extremely amplified families, which altogether represent the great majority of the entire satDNA content [13]. Interestingly, a similar situation is observed in the Heteroptera species *Holhymenia histrio*, where the most abundant satDNA family represents 14% of the genome, while all satDNA are 17% of the genome [15].

**Figure 1:**
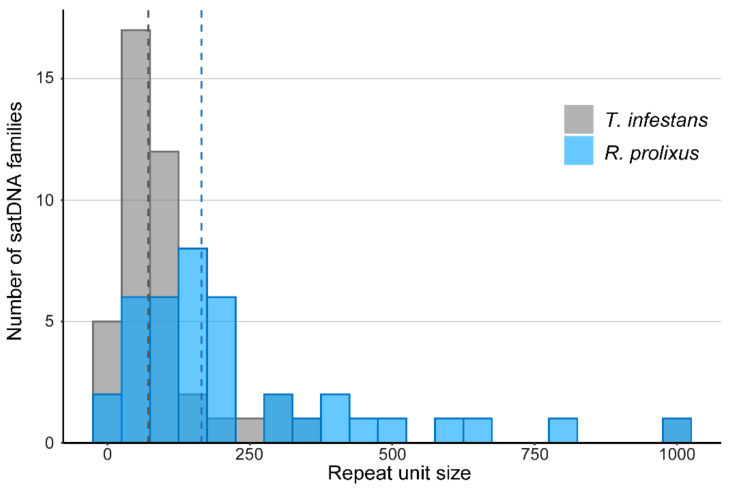
Repeat unit size distribution on *Rhodnius prolixus* and *Triatoma infestans*. Dashed lines represent median value of repeat unit size for *R. prolixus* (163 bp) and *T. infestans* (72 bp).

The size of the repeat units showed great variation, from 31 bp (RproSat29-31) up to near 1 kb (RproSat22-980), regardless of the telomeric and (GATA)_n_ repeats (Figure 1). Most of the satDNA families have repeat units smaller than 300 bp, although the most frequent sizes were between 100 and 200 bp (median = 163 bp). This is different to the size pattern found in *T. infestans,* in which most of the satDNA families have repeat units smaller than 100 bp (median = 72 bp) (Figure 1). The A + T content of satDNA family sequences ranges between 27.8% (RproSat27-187) and 83.3% (RproSat11-198) (Table 1, Supplementary Table S1). According to our sequencing data, the A + T content of paired-end reads is 65.37%, indicating that satDNA sequences, with a mean of 65.7%, are not especially enriched in A + T. Furthermore, the A + T richness of *R. prolixus* satellitome is just slightly higher than the *T. infestans* one (range of 44.3–81.6%, mean of 64.3%) [13]. Nucleotide divergence of satDNA families in *R. prolixus* is also similar to other satellitomes. Ranging between 0.88% (RproSat32-59) and 28.28% (RproSat13-293), the satellitome divergence of *R. prolixus* shows a median value of 10.03% (Table 1), similar to that described for the grasshopper *Eumigus monticola* (9.21%), the cricket *Gryllus assimilis* (9.3%) or the fish *Megaleporinus macrocephalus* (10.89%) [18,19,23]. Notwithstanding, this divergence value is double that of the satellitome divergence of the beetle *Hippodamia variegata* (5.75%) [21].

**Figure 2:**
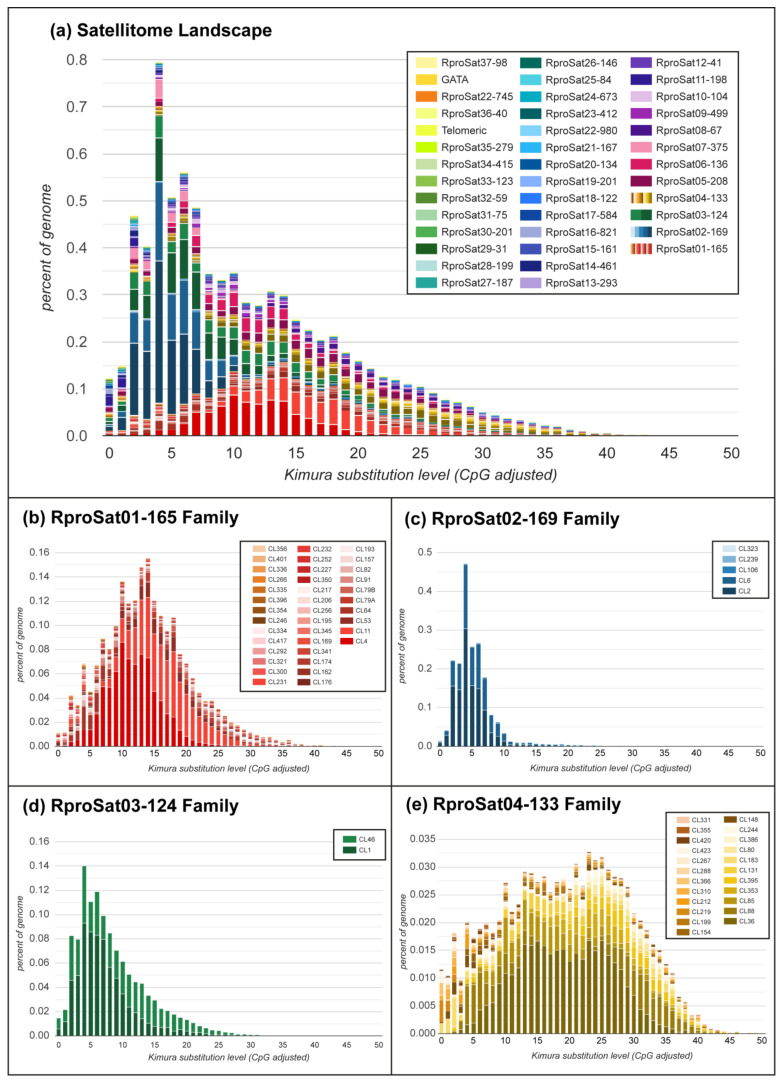
(**a**) Satellitome landscape of the satDNA families in *Rhodnius prolixus*. In the landscape, abundance vs. Kimura divergence from satDNA consensus sequences is plotted. (**b**–**e**) Landscape of most abundant satDNA families divided by their subfamilies. The order of satDNA families and subfamilies are in order of their position on a stacked histogram.

When *R. prolixus* satellitome distribution of abundance vs. divergence with respect to each consensus sequence was analyzed, a right-skewed distribution was obtained with a peak below 5% divergence and a long tail (Figure 2a). Amplification and homogenization processes, inversely related to divergence, and point mutation, directly related to divergence [25,53], are the main forces of the satDNA evolution. Bearing that in mind, the satellitome landscapes are very informative about the satDNA structure on the genome, where more homogeneous satDNA families will present narrow and high distribution while dispersed satDNA families will show wide and flattened distributions.

**Figure 3:**
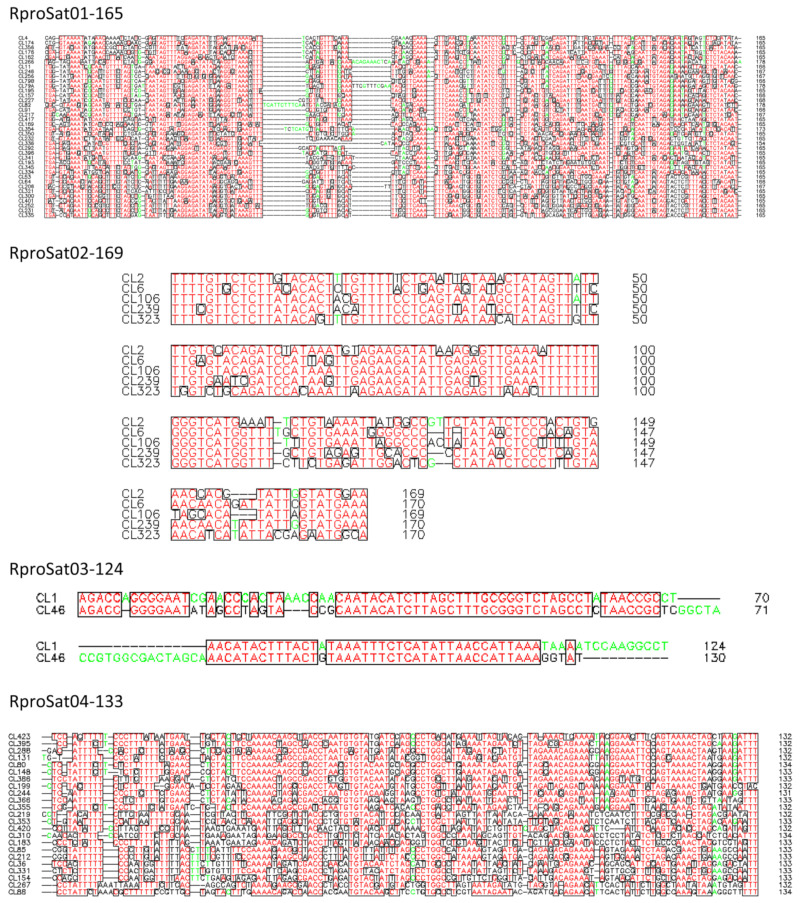
Consensus sequences alignments of the different subfamilies found for the most abundant satDNA families in *Rhodnius prolixus*. Boxed red letters correspond with conserved position among sequences. Less conserved positions are indicated with green and black letters.

**Figure 4:**
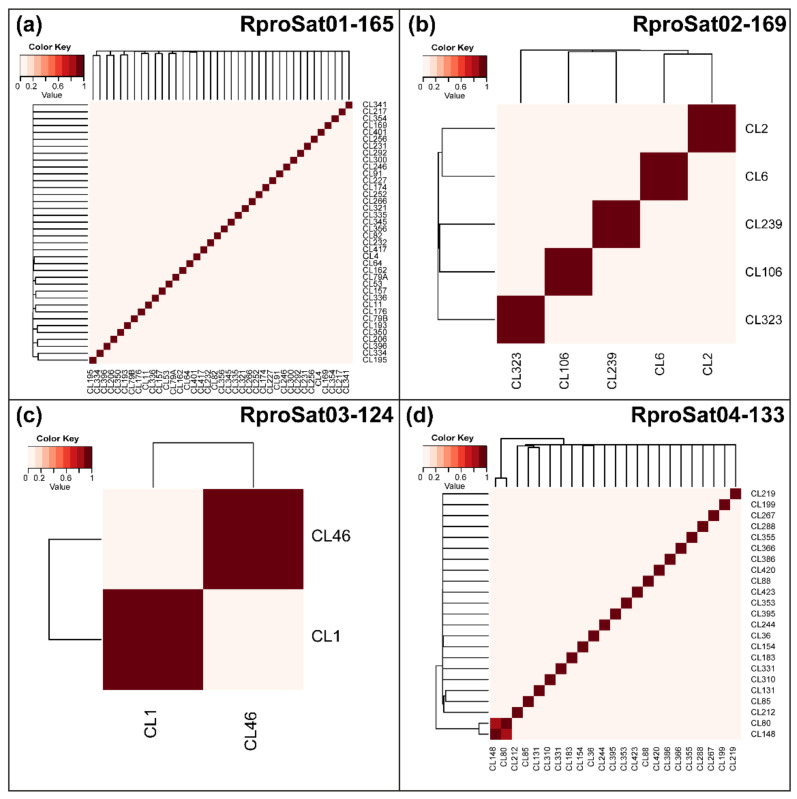
Heatmap representing the fraction of *Rhodnius prolixus* genome scaffolds shared by two subfamilies: (**a**) RproSat01-165, (**b**) RproSat02-169, (**c**) RproSat03-124 and (**d**) RproSat04-133. The figure shows that almost no subfamilies coincide in the same scaffold.

**Figure 5:**
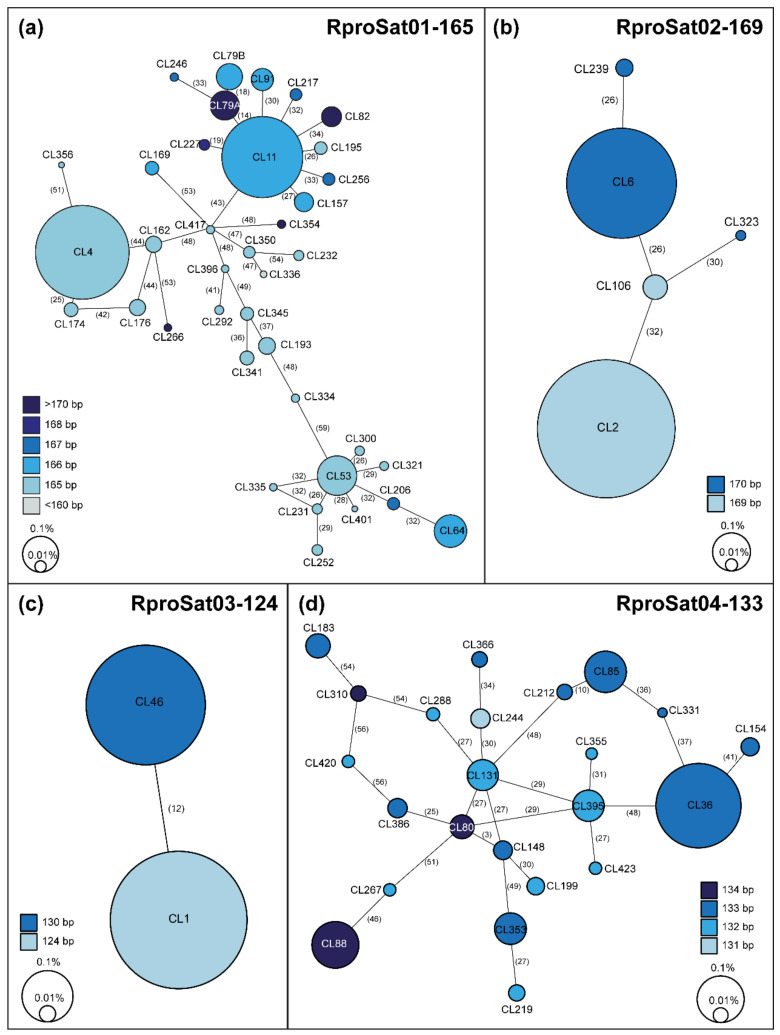
Minimum spanning networks for the four satDNA families forming superclusters in *Rhodnius prolixus*: (**a**) RproSat01-165, (**b**) RproSat02-169, (**c**) RproSat03-124 and (**d**) RproSat04-133. Numbers between brackets are the mutational steps. Each circle corresponds to a RepeatExplorer2 cluster or subfamily, where the size is proportional to its abundance in the genome. Colors denote the consensus monomer length.

Clustering analysis revealed that the most abundant satDNA families formed superclusters. A supercluster is a set of clusters of the same repetitive DNA [16]. The highest numbers of clusters forming a supercluster were found for RproSat01-165 and RproSat04-133, with 38 and 23 clusters, respectively (Table 1). Each cluster generated a different consensus sequence, which was named with the original cluster name given in the RepeatExplorer2 output, for instance, CL1, CL2, etc. Figure 3 shows the alignment of the consensus sequences from each cluster of these satDNA families. If the different variants of the satDNA family were clustered on different arrays in the genome, they would represent true subfamilies. However, if the different variants of a satDNA family were mixed, they would not belong to different subfamilies, although they have been separated and assigned to different clusters by RepeatExplorer2. In order to test these two alternative hypotheses, we have analyzed the presence of each cluster consensus sequence in the *R. prolixus* genome assembly [1]. Searches in the assembled genome showed that the great majority of the contigs or scaffolds of the assembled genome contain only one of the variants (Figure 4), suggesting the existence of true satDNA subfamilies for the four most abundant satDNA families. The only exception was found in the RproSat04-133, where two of the sequence variants appeared together in more than 80% of the scaffolds (Figure 4d, clusters 80 and 148). This may be an artefact generated by the high similarity between the consensus sequences of these two subfamilies (over 97%), which makes it difficult to discriminate them from each other, and hence hampers the analysis. Additionally, minimum spanning networks were generated to analyze satDNA subfamilies’ relationships within each family. The most complex networks correspond to satDNA families with higher amounts of members, RproSat01-165 and RproSat04-133. In the RproSat01-165 family, the most abundant subfamilies are close in the network, with the CL11 subfamily acting as a network node (Figure 5a). In the RproSat04-133 family, the network is more complex and reticulate than in the RproSat01-165 one, coinciding with a higher Kimura divergence and broader and more flattened landscape (Figure 2e and Figure 5d).

As discussed above, repetitive DNAs may not be well-represented in assembled genomes. In order to know how well-represented the satellitome is on *R. prolixus* genome assembly, pseudo reads were generated from it to estimate their abundance and divergence with RepeatMasker (Supplementary Table S1). Thereupon, just 5.6% of the assembled genome corresponded to satDNA sequences, and four families detected by RepeatExplorer2 were missing (Supplementary Table S1). This result is significantly lower than that obtained by us (8.05%) from the unassembled reads. Nevertheless, it is not possible to be sure if these repeated DNA sequences consist in a great part of those which were left out from the genome assembly, or if they may be collapsed. In any case, this shows the importance of the knowledge of satDNA abundance prior to the sequencing assembly of a Triatominae genome. Discrepancies of satDNA abundance estimations between our analysis and assembled genome are smaller in *R. prolixus* than in other insects [54]. This might be due to the fact that the *R. prolixus* satDNAs are scattered on the genome organized into small arrays. In species with large heterochromatic blocks, genome assembly will cover until those block edges, so intern repeats could not be assembled and the amounts of discarded sequences will be higher, underestimating the amount of satDNA.

Available NCBI *R. prolixus* raw reads from genomic DNA were also included in the analysis (SRR6749969, SRR6749971, SRR6749972 and SRR6749978). It is important to note that *R. prolixus* belong to a complex of cryptic species with the ability to hybridize. This issue could lead to erroneous interpretation of the data, as it has been recently revised [55]. Hence, the correct species classification was checked using the ribosomal internal transcribed spacer 2 (ITS-2) region of each dataset (Supplementary Figure S3). The amount of satDNA in these four archives of sequencing data ranges between 8.81% and 9.62%, closer to our estimate (8.05%) than that obtained from the assembled genome (5.6%) (Supplementary Table S1, Figure S1). Notwithstanding, as already shown on other insect species [13,14,20], variations in the amount—and even the absence—of some satDNA families were found between individuals. In spite of this, the general aspect of the repeat landscape for each genome was conserved (Supplementary Figure S1b–e).

**Figure 6:**
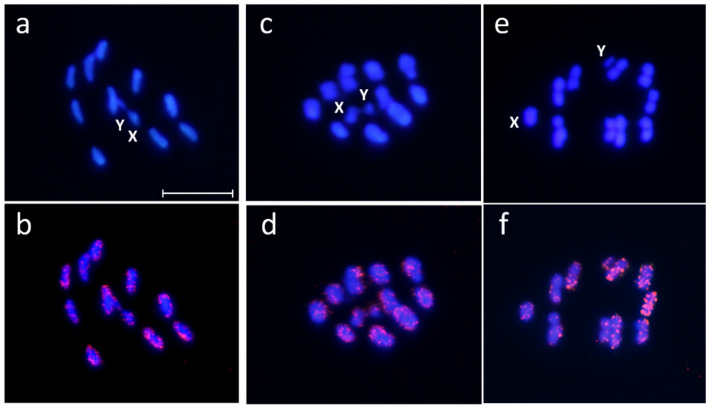
Chromosomal location of most abundant satDNA families of *Rhodnius prolixus*. Male meiotic metaphases stained with DAPI (**a**,**c**,**e**). Merged images of FISH with RproSat01-165 probe (**b**), RproSat02-169 probe (**d**) and RproSat03-124 (**f**). Scale bar = 10 µm.

Chromosomal location of the most abundant satDNA families (over 1% of the genome) was performed by fluorescence in situ hybridization (FISH). Hybridization with satDNA probes showed disperse signals over euchromatin of all chromosomes, autosomes and both sex chromosomes (Figure 6). These cytogenetic results are in agreement with the molecular results. The analysis of the satDNA subfamilies in the assembled *R. prolixus* genome showed that these satDNA were present in a high number of scaffolds (Supplementary Figure S4), but the number of monomers in each scaffold was low, most of them with less than 50 repeats (Supplementary Figure S5). This hybridization pattern is similar to that found for other less abundant families, such as the four satDNA families shared between *R. prolixus* and *T. infestans* [11]. All data support the different composition of the heterochromatic Y chromosome between Triatomini and Rhodniini tribes [40,41]. The satDNA family TinfSat01-33 and (GATA)_n_ repeats are the main components of the *T. infestans* Y chromosome heterochromatin, and no other satDNA families were present in this chromosome [41]. On the contrary, the *R. prolixus* Y chromosome contains several satDNA families, in the same way as autosomes and the X chromosome. In *Triatoma* species, (GATA)_n_ repeats are especially accumulated on the Y chromosome, and these repeats seem to be the only repetitive DNA shared by the Y chromosomes of this genus [40,41]. However, (GATA)_n_ repeats are not abundant in the *R. prolixus* genome, and FISH with (GATA)_n_ repeats showed no signals on the Y chromosome (data not shown).

**Figure 7:**
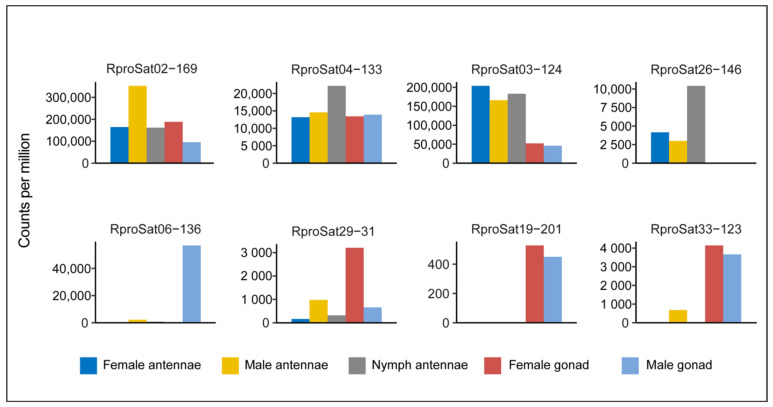
Examples of the four transcription patterns shown by satDNA families of *R. prolixus* in different tissues.

The possible role of transcripts of satDNA has been questioned in the past, but accumulation of evidence has changed that view. Currently, it is widely accepted that satellite non-coding RNAs might have functions in different cellular contexts, such as cancer, stress response, development or cell proliferation [32,33,56]. Hence, we have analyzed whether satDNA families of *R. prolixus* are transcribed in different tissues. Samples from two available RNA-seq experiments from tissues were selected. The first one was an RNA-seq analysis of antenna, the main chemosensory structures in insects, performed in nymph, male and female (SRX1011796, SRX1011778 and SRX1011769, respectively), and the second one, an RNA-seq analysis performed in female and male gonads (SRX6380683 and SRX6380682, respectively). Due to the taxonomy identification issue commented on above, the ITS2 region sequence was obtained from each dataset in order to check the correct species classification (Supplementary Figure S3). After mapping dataset reads to our satDNA consensus sequences, satDNA families poorly represented were discarded and read count was normalized. Library preparation for RNA-sequencing is highly decisive for results. Therefore, library selection from antenna was random, while enrichment for messenger RNA sequences was applied at gonads library. Bearing that in mind, comparison between experiments should be made with caution since non-coding RNA could probably be under-represented on gonads samples, and satDNA transcription could be underestimated. We found that 33 satDNA families were transcribed at least in two samples (Figure 7 and Supplementary Figure S6, Table S2), showing four different patterns. The first pattern would correspond to satDNA families transcribed in all studied tissues (antenna, ovaries and testis), although with different proportions, such as RproSat02-169 and RproSat04-133. The second pattern would represent satDNA families highly transcribed in antenna, with less or no transcription in gonads, such as RproSat03-124 and RproSat26-146. The third pattern would correspond to satDNA families highly transcribed in gonads, such as RproSat19-201 and RproSat33-123. Finally, the fourth pattern would belong to satDNA families highly transcribed in one gonadal tissue only, such as RproSat06-136 (testis) and RproSat29-31 (ovaries). Additionally, the transcription level of satDNA families appears to be significantly correlated with their abundance, whether combined transcription (Spearman’s correlation r_s_ = 0.52, *p* = 0.002) or tissue transcription (Supplementary Table S3) is considered. However, one exception should be pointed out, the Rpro34-415 family. This family, which represents only 0.003% of the genome, showed transcription levels similar to the most abundant families, being higher at male antennae and gonads. Together, those results indicated the satellitome is generally expressed in *R. prolixus*, although each family is transcribed at a different level and at a different pattern, suggesting that satDNA transcription could have a specific role in those tissue environments. Satellite DNA transcription is an accepted feature, as we commented on above, and it has been seen before in other insects, such as Coleoptera [57,58,59], Hymenoptera [60,61,62], Orthoptera [19], Lepidoptera [63] or Diptera [33,34,64]. In *D. melanogaster*, Mills et al. [34] found that satDNA derived from (AAGA)_n_ tandem repeat is highly transcribed at neuron and testis, being necessary for male fertility. Another *D. melanosgaster* satDNA, the 1.688 satDNA family, contains a member with a dense X-linked distribution (1.688^X^), which plays an important role in marking the X chromosome during dosage compensation [65]. In males, the small interfering RNAs generated from 1.688^X^ sequences promote X localization of the male-specific lethal complex, which increases X-linked gene expression by modification of chromatin [38,63]. In *D. buzzatti* and *D. mojavensis*, satDNA families pBuM and CDSTR198 were transcribed, particularly in pupae and male tissues, even when both satDNAs have different genomic environment (heterochromatin and euchromatin, respectively) [64]. Outside of insects, satDNA has proven their importance. For instance, in humans, SATIII is associated with cell response to stress, recruiting RNA-processing factor and downregulating cellular transcription [32]. Our findings suggest that satDNA transcription might have functionality on the *R. prolixus* genome and open the door to future studies to address whether those satDNAs contribute to gene regulation or chromatin modulation.

## 3. Materials and Methods

### 3.1. Samples, DNA Extraction and Chromosome Preparation

Domestic *R. prolixus* individuals were collected from Colombia (Department Casanare, Municipality Yopal). DNA extraction for sequencing was performed from the head of an adult male using the NucleoSpin Tissue kit (Macherey-Nagel Co., Düren, Germany). For cytogenetic analysis, adult males were dissected, and the testes were fixed in an ethanol–glacial acetic acid mixture (3:1) and stored at −20 °C. Squashes were made in a 50% acetic acid drop, coverslips were removed after freezing in liquid nitrogen and the slides were air-dried and stored at 4 °C [13].

### 3.2. DNA Sequencing and Graph-Based Clustering of Sequencing Reads

Low-coverage sequencing was performed using the DNBseq^TM^ sequencing platform at BGI, Hong Kong, which yielded 1.2 Gbp of PE150 reads. Raw reads were first quality trimmed with Trimmomatic [66]. Fastq files were modified, i.e., discarded reads containing Ns, fastq to fasta, with the FastX toolkit (http://hannonlab.cshl.edu/fastx_toolkit, accessed on 3 May 2021). Sequences corresponding to mitochondrial DNA were eliminated from the repeat analysis. NCBI-deposited genomes’ raw reads were downloaded using prefetch and fastq-dump tools (SRR6749969, SRR6749971, SRR6749972 and SRR6749978).

As *R. prolixus* species determination can be tricky, the ITS2 ribosomal region sequence was extracted from raw data by mapping with bbmap (sourceforge.net/projects/bbmap/, accessed on 3 May 2021) against the *R. prolixus* ITS2 sequence (DQ118978). A phylogenetic maximum likelihood tree was performed using all the available sequences from other *Rhodnius* species from GenBank. Alignment was performed with MAFFT [67] and a phylogenetic tree was constructed using the ML method in RaxML [68]. The tree was edited with iTOL [69].

Graph-based clustering was performed using the RepeatExplorer2 pipeline, which includes the TAREAN analysis, on the Galaxy portal environment (https://repeatexplorer-elixir.cerit-sc.cz, accessed on 3 May 2021). A set of six million paired-end reads were randomly selected for clustering analysis. Clusters containing satDNAs were identified based on the graph topology with sphere or ring-like shape. For each candidate cluster, we chose the longest and the highest coverage contig assembled by RepeatExplorer2 to generate a dot plot with the Dotmatcher tool (http://emboss.bioinformatics.nl/cgi-bin/emboss/dotmatcher/, accessed on 3 May 2021). Afterwards, contigs were separated in monomers to align them using MAFFT and generate a consensus sequence. All satDNA consensus sequences were submitted to NCBI (Acc. Numbers MW827131-MW827167). When all satDNA clusters where annotated and a monomer consensus was obtained, similarity between them was tested using the Basic Local Alignment Search Tool (BLAST), with blastn and –e 0.001 options. Additionally, divergence and abundance for each satDNA were calculated using RepeatMasker (http://www.repeatmasker.org, accessed on 3 May 2021) with “-a” option and the RMBlast search engine. For this, we randomly selected a million reads and aligned against the total collection of satDNA dimers or monomer concatenations of approximately 200 bp length. We estimated the average divergence and generated a satellite landscape considering distances from the sequences applying the Kimura 2-parameter model with the perl script calcDivergenceFromAlign.pl and createRepeatLandscape.pl from the RepeatMasker suite. Subfamilies’ consensus alignments were plotted with Prettyplot (https://www.bioinformatics.nl/cgi-bin/emboss/prettyplot, accessed on 3 May 2021) and minimum spanning networks were built according to the pairwise distance of subfamilies’ consensus sequences and considering the relative abundances using PopART v1.7 [70,71].

### 3.3. Transcription of Satellite DNA

We downloaded *R. prolixus* RNA-seq data from two bio-projects from the NCBI database: an antenna transcriptome project (SRX1011796, SRX1011778, SRX1011769) and a sex differentiation of gonad transcription project (SRX6380683, SRX6380682). To check that insects used were *R. prolixus,* ribosomal ITS-2 spacer reads were extracted by mapping reads from all sets with bbmap (sourceforge.net/projects/bbmap/, accessed on 3 May 2021) against the *R. prolixus* ITS-2 spacer (DQ118978).

Raw RNA-seq data from all tissues were mapped to each satDNA consensus using bbmap to obtain the output as a sam file. We used the same satDNA dimers or monomer concatenations as used for abundance analysis as references. The aligned reads were counted using samtools [72]. Read counts were analyzed in R base version 4.0.1 [73] using the edgeR package [74]. In brief, read counts from all tissues were normalized to counts per million (CPM) and filtered satDNAs with more than 50 CPM in at least 2 samples. Correlation between satDNA transcription and abundance was analyzed by means of Spearman correlation and graphs were obtained with the ggplot2 package [75] in R.

### 3.4. Cytogenetic Mapping

The consensus sequences of the most abundant satDNA families, over 1% of the genome, were used to design a set of oligonucleotides (Supplementary Table S4). These labeled oligonucleotides were used as probes (final concentration of 5 ng/mL in 50% formamide) to perform fluorescence in situ hybridizations (FISH) according to the procedure described by Palomeque et al. [76] and Pita et al. [13]. The fluorescent immunological detection was carried out using the avidin-FITC/anti-avidin-biotin system with three amplification rounds. Slides were mounted in Vectashield–DAPI (Vector Laboratories, Burlingame, CA, USA). DAPI, in the antifade solution, was used to counterstain the chromosomes. Images were taken with a BX51 Olympus^®^ fluorescence microscope (Olympus, Hamburg, Germany) equipped with a CCD camera (Olympus^®^ DP70) and processed using Adobe^®^ Photoshop^®^ software.

### 3.5. Rhodnius Prolixus Genome Assembly satDNA Families Searches

*Rhodnius prolixus* genome assembly was downloaded from VectorBase (https://vectorbase.org/vectorbase/app/, accessed on 3 May 2021), which is the same as that available in GenBank: GCA_000181055.3.

To include this data in the RepeatExplorer analysis, a simulated Illumina paired-end 150 bp reads run was performed using ART [77].

The search for the described satDNA families was carried on with a Basic Local Alignment Search Tool (BLAST) analysis. Only those hits with 90% of query coverage per HSP and a 90% identity were taken into account. To render the heatmaps, BASH text-processing tools and R base version 4.0.1 [73] with gplots [78] and RColorBrewer [79] packages were employed. The same BLAST results were used to evaluate the amount of satDNA families by contig or scaffold, and figures were obtained with the ggplot2 package [74] in R.

## Figures and Tables

**Table 1:** Data of the satDNA families found in *Rhodnius prolixus*: genome abundance (%), length of the repeat unit, A + T content and divergence (%). The number of clusters in the RepeatExplorer2 analysis is also shown. GenBank accession numbers: MW827131 to MW827167.

Name	No. of RE Clusters	Genome Proportion	Repeat Unit Length (bp)	A + T Percentage	Kimura Divergence (%)
RproSat01-165	38	2.13%	165	72.1%	13.42%
RproSat02-169	5	1.94%	169	69.2%	9.81%
RproSat03-124	2	1.18%	124	62.1%	8.81%
RproSat04-133	23	0.861%	133	66.2%	17.38%
RproSat05-208	1	0.460%	208	53.4%	15.48%
RproSat06-136 ^1^	1	0.320%	136	62.5%	12.42%
RproSat07-375	1	0.200%	375	68.5%	7.77%
RproSat08-67	1	0.194%	67	61.2%	20.58%
RproSat09-499	1	0.107%	499	71.1%	12.20%
RproSat10-104	1	0.085%	104	62.5%	10.10%
RproSat11-198	1	0.073%	198	83.3%	2.23%
RproSat12-41	1	0.069%	41	58.5%	10.03%
RproSat13-293 ^1^	-	0.067%	293	61.1%	28.28%
RproSat14-461	1	0.062%	461	64.2%	6.19%
RproSat15-161	1	0.052%	161	60.9%	8.38%
RproSat16-821	1	0.044%	821	65.3%	6.83%
RproSat17-584	1	0.027%	584	64.7%	11.71%
RproSat18-122	1	0.019%	122	63.1%	25.27%
RproSat19-201	1	0.019%	201	76.1%	19.52%
RproSat20-134	1	0.017%	134	68.7%	16.23%
RproSat21-167	1	0.016%	167	71.3%	16.25%
RproSat22-980	2	0.013%	980	69.4%	4.75%
RproSat23-412	1	0.010%	412	75.2%	5.59%
RproSat24-673	1	0.009%	673	70.0%	2.89%
RproSat25-84 ^1^	-	0.009%	84	65.5%	25.75%
RproSat26-146	1	0.009%	146	63.7%	3.55%
RproSat27-187	1	0.008%	187	27.8%	13.06%
RproSat28-199	1	0.008%	199	53.8%	12.72%
RproSat29-31	1	0.006%	31	77.4%	11.21%
RproSat30-201	1	0.006%	201	61.2%	3.02%
RproSat31-75	1	0.005%	75	68.0%	2.64%
RproSat32-59	1	0.004%	59	59.3%	0.88%
RproSat33-123	1	0.003%	123	78.0%	0.96%
RproSat34-415	1	0.003%	415	72.8%	5.15%
RproSat35-279	1	0.003%	279	60.6%	5.71%
RproSat36-40	1	0.003%	40	70.0%	4.17%
RproSat37-98 ^1^	-	0.0005%	98	69.4%	6.96%
Telomeric repeat ^1^	-	0.003%	5	60.0%	13.9%
(GATA)_n_ repeat ^1^	-	0.001%	4	75.0%	10.1%
Total		8.05%			
Mean			235.23	65.72%	10.56%
SD			223.13	9.10%	6.91%
Median			165	65.50%	10.03%

^1^ SatDNA families also present in the *Triatoma infestans* genome.

## Data Availability

All satDNA consensus sequences were submitted to NCBI (Acc. Numbers MW827131-MW827167).

## Supplementary Materials

The following are available online at https://www.mdpi.com/article/10.3390/ijms22116052/s1. Figure S1: Repeat composition of *Rhodnius prolixus* genome obtained from RepeatExplorer2 annotation (a). Satellitome landscape of the satDNA families in NCBI samples (b–d). Figure S2: Alignment of consensus sequences of satDNA families with similarity: RproSat07-375 and RproSat09-499 (a), and RproSat22-980 and Rpro24-675 (b). Similar positions are marked with red font and red boxes indicate regions with high similarity. Sequences are numbered according to the original position of consensus sequences. Figure S3: ML phylogenetic tree depicting the position of samples used in our study within *Rhodnius prolixus*. Figure S4: Number of scaffolds where subfamilies are distributed on *Rhodnius prolixus* genome assembly. Figure S5: Monomer distribution of most abundant satDNA families in *Rhodnius prolixus* assembled genome. Histograms relate number of scaffolds and monomers. For all families, long arrays are present on few scaffolds. Figure S6: SatDNA transcription in different tissues. Table S1: Estimations of genome abundance and nucleotide divergence of satDNA families and subfamilies found in *Rhodnius prolixus* genome sequenced in this work (Rpro) and in pseudo-reads generated from *Rhodnius prolixus* genome assembly (GCA_000181055.3) (RproSim). Subfamilies’ names are followed by the size of their consensus sequence. Table also shows the same data for available NCBI *Rhodnius prolixus* raw reads from genomic DNA: Rpro1 (SRR6749969), Rpro2 (SRR6749971), Rpro3 (SRR6749972) and Rpro4 (SRR6749978). Table S2: SatDNA transcription on different tissues. Combined column refers to sum of all samples’ transcription for each satDNA family. Table S3: Results of Spearman correlation between satDNA transcription and abundance in different samples. Table S4: Designed oligonucleotides for three main satellite DNA families of *Rhodnius prolixus*.
