# Supplementary material for: Satellitome Analysis of Rhodnius prolixus, One of the Main Chagas Disease Vector Species
Source: Int J Mol Sci. 2021 Jun 3;22(11):6052. doi: 10.3390/ijms22116052 (PMC8199985; doi:10.3390/ijms22116052)
Supplement: Supplementary file 1 [file ijms-22-06052-s001.zip › ijms-1229100-supplementary.pdf]

Supplementary Material

Satellitome analysis of *Rhodnius prolixus*, one of the main Chagas disease vector species

Eugenia E. Montiel, Francisco Panzera, Teresa Palomeque, Pedro Lorite, Sebastián Pita

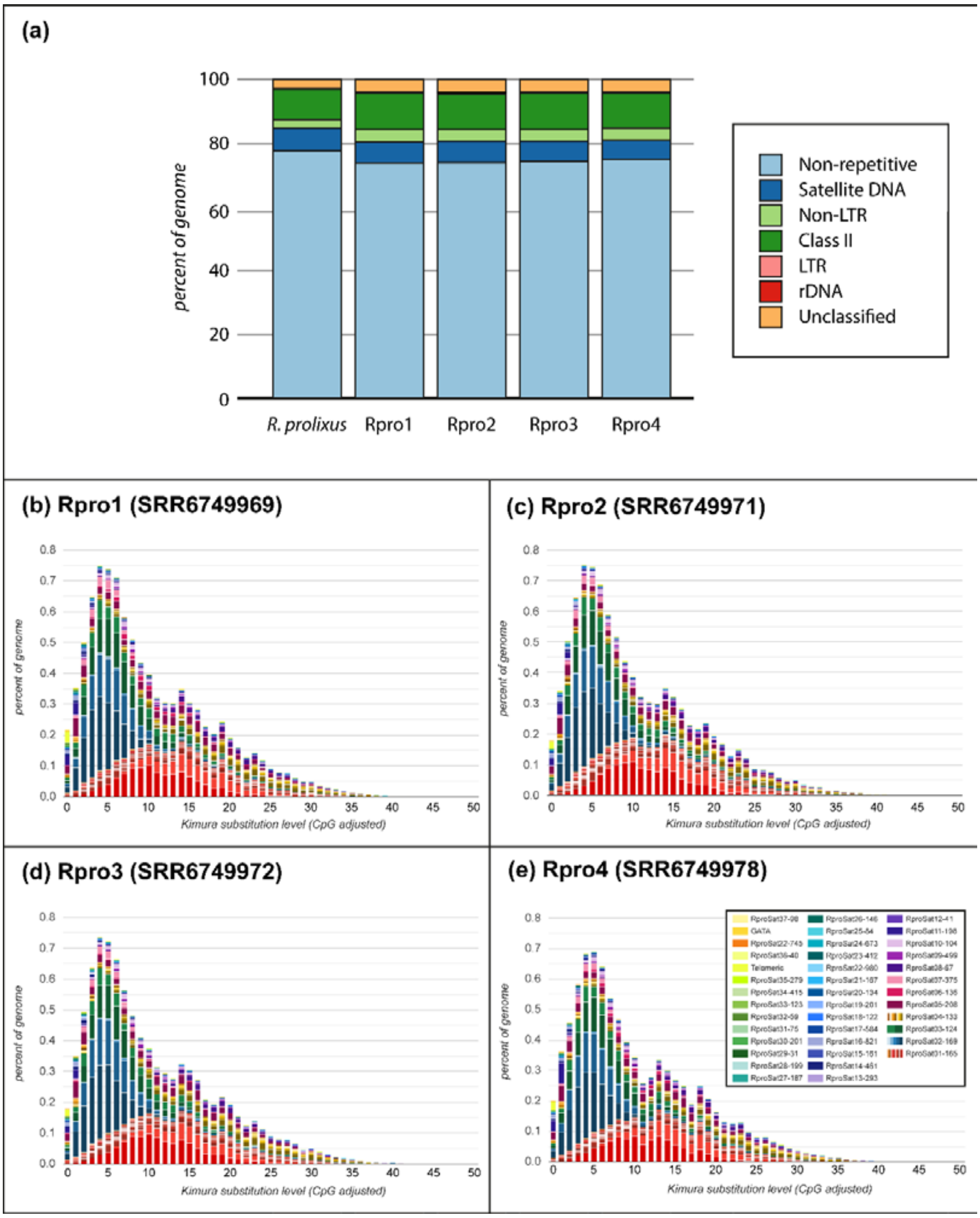

Figure S1. Repeat composition of *Rhodnius prolixus* genome obtained from RepeatExplorer annotation **(a)**. Satellitome landscape of the satDNA families in NCBI samples **(b-e)**.

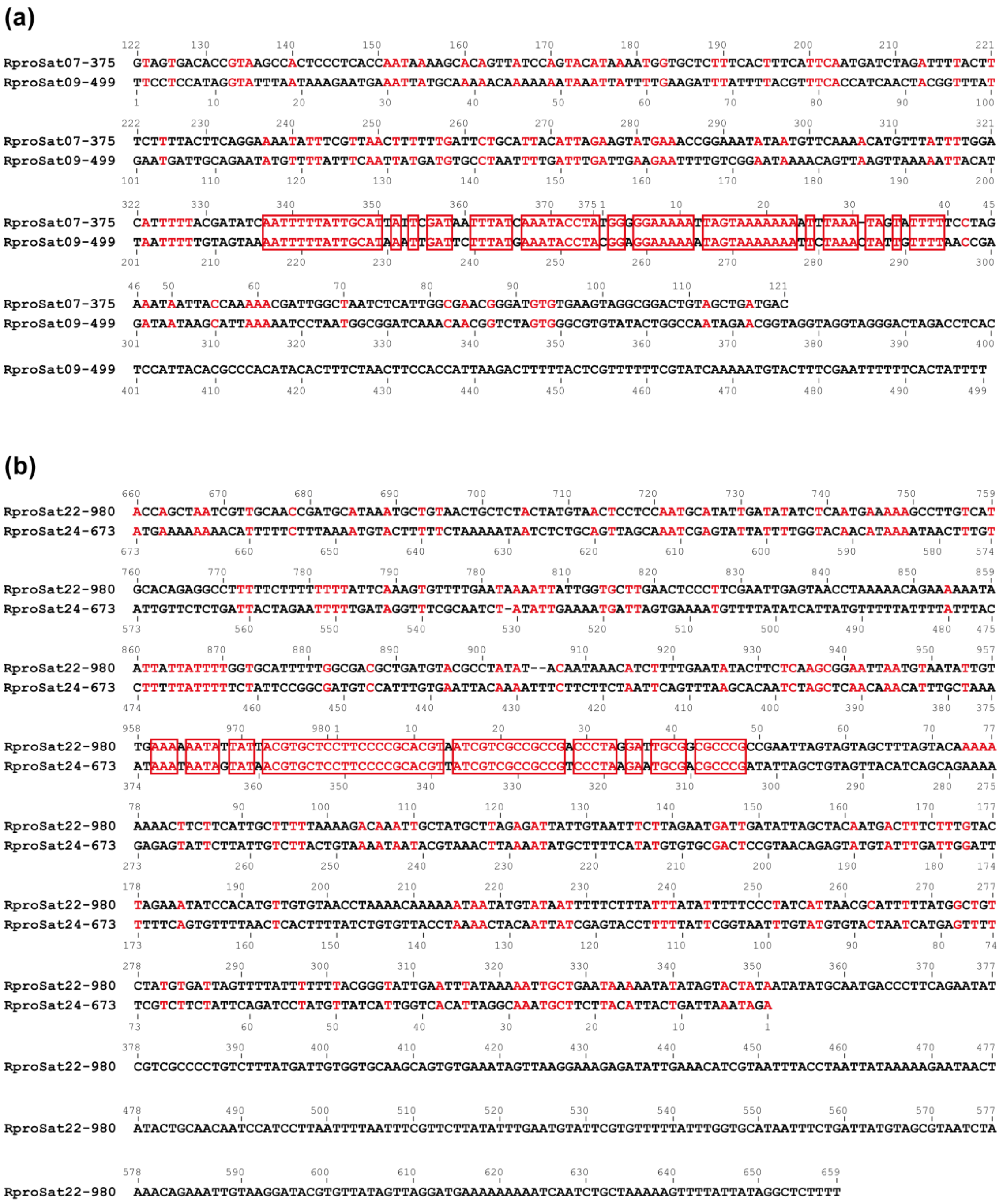

Figure S2. Alignment of consensus sequences of satDNA families with similarity: RproSat07-375 and RproSat09-499 (a), and RproSat22-980 and Rpro24-675 (b). Similar positions are marked with red font and red boxes indicate regions with high similarity. Sequences are numbered according to the original position of consensus sequences.

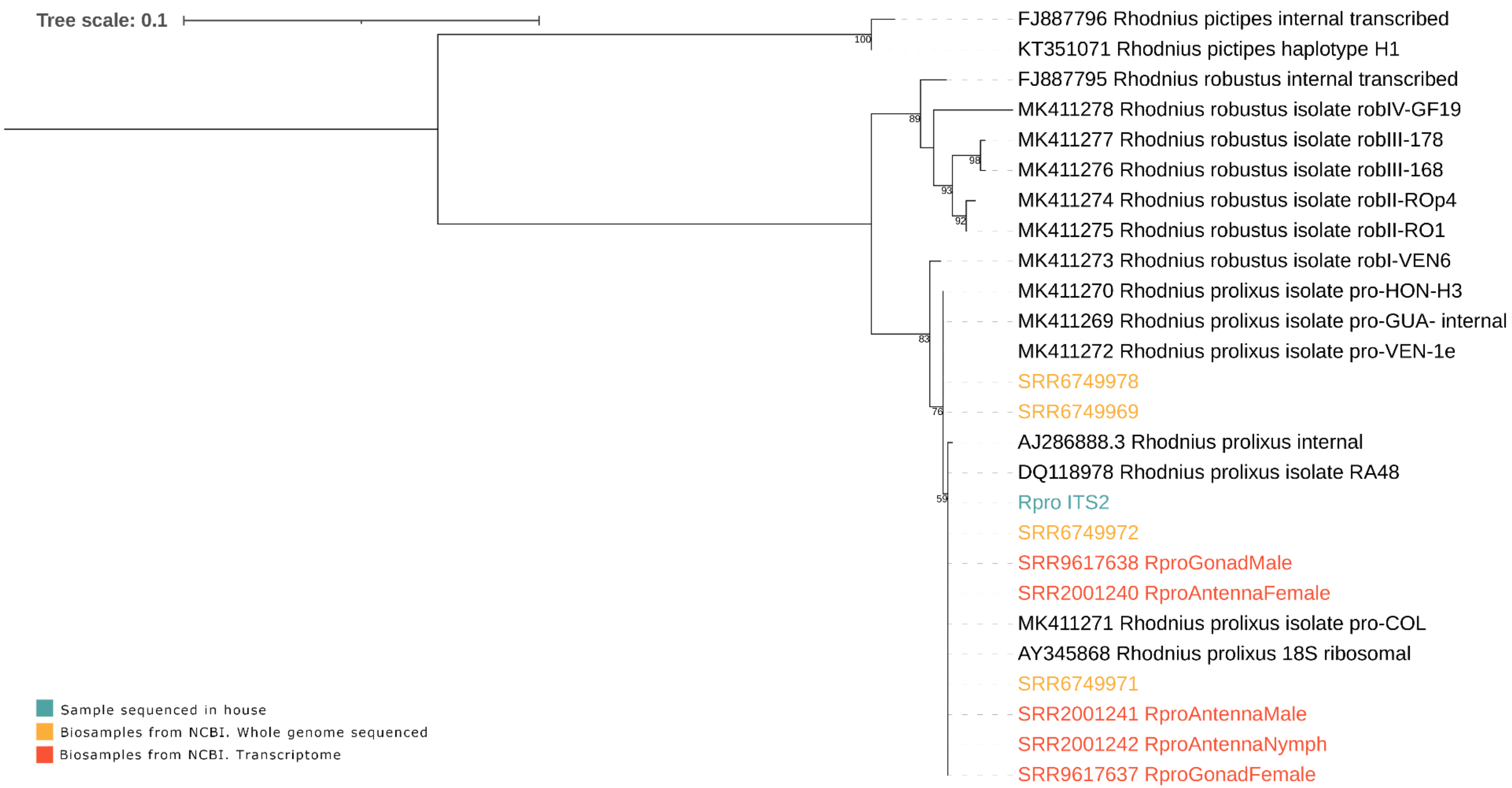

Figure S3. ML phylogenetic tree depicting the position of samples used in our study within *Rhodnius prolixus*.

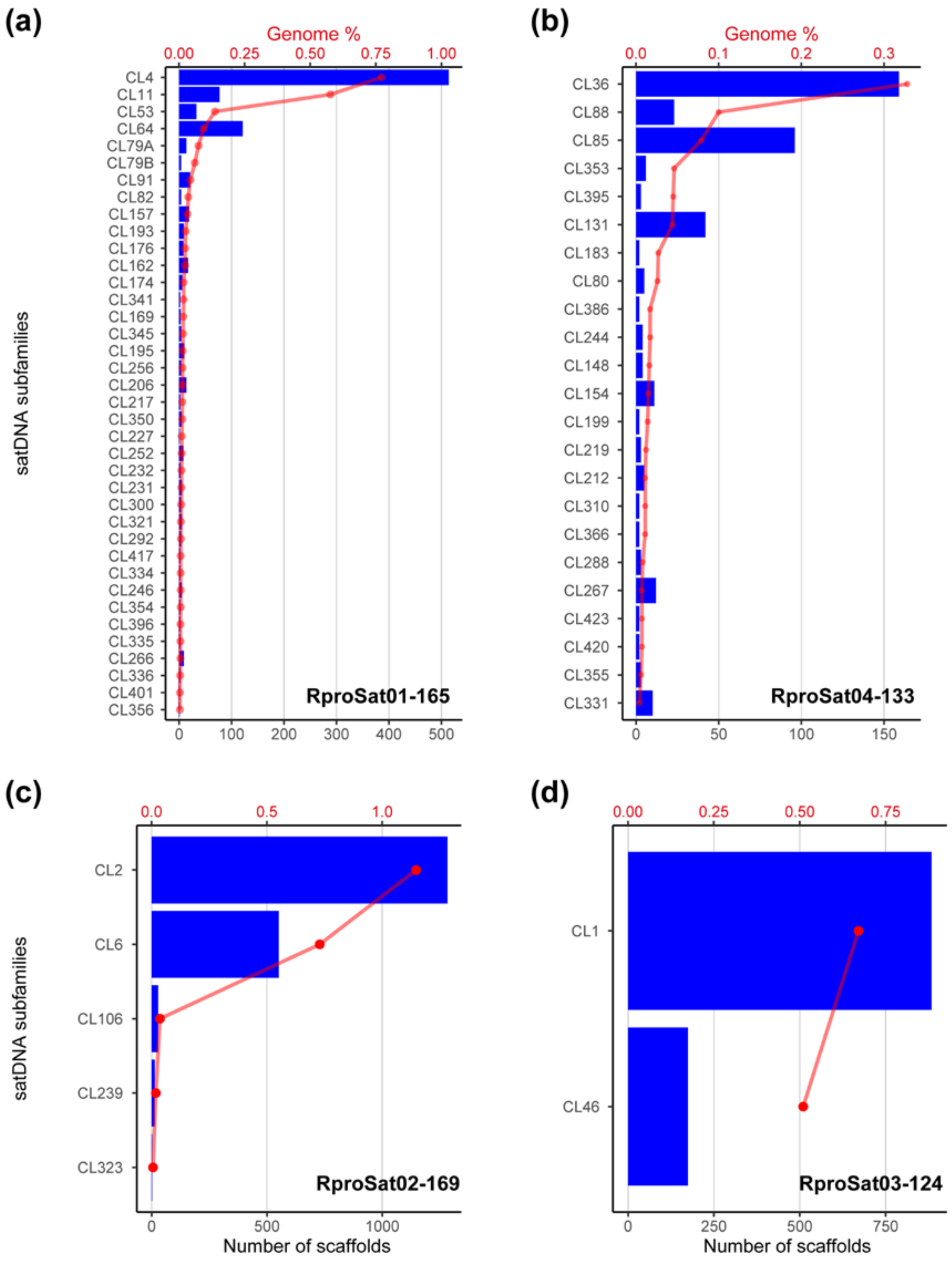

Figure S4. Number of scaffolds where subfamilies are distributed on *Rhodnius prolixus* genome assembly.

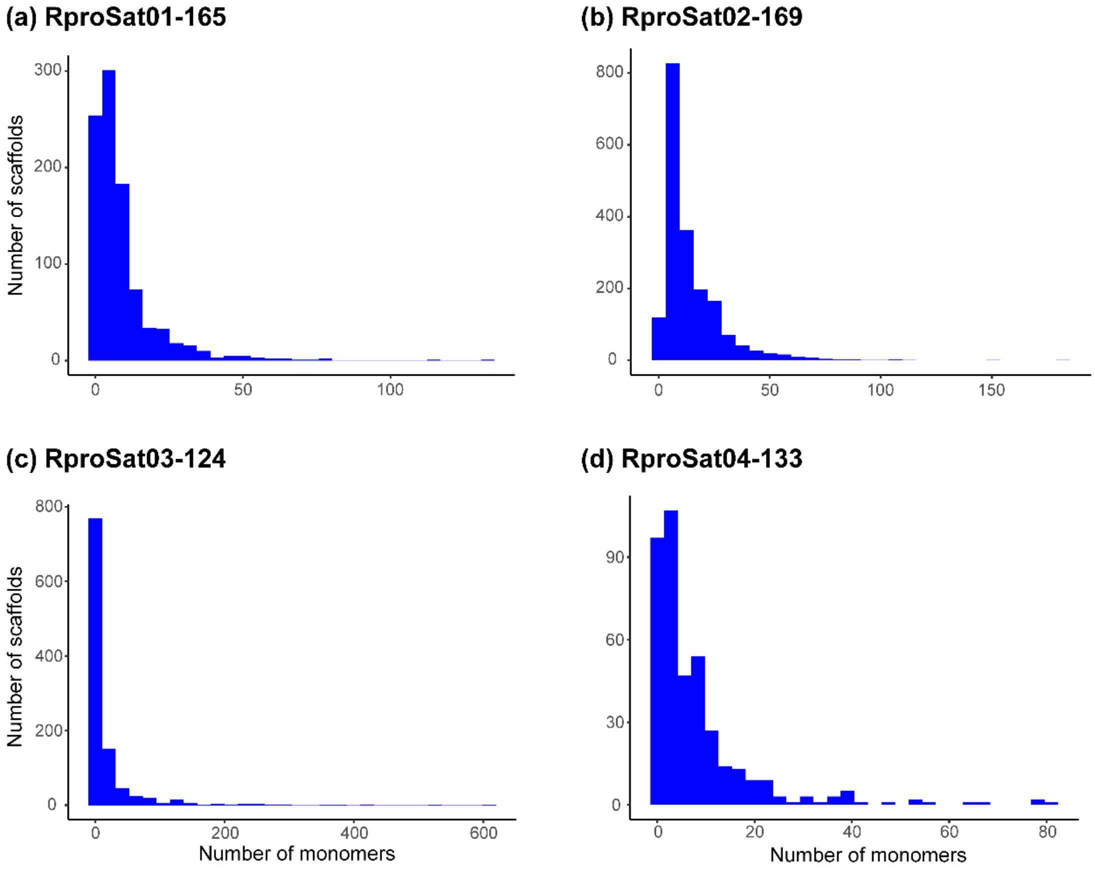

Figure S5. Monomer distribution of most abundant satDNA families in *Rhodnius prolixus* assembled genome. Histograms relate number of scaffolds and monomers. For all families, long arrays are present on few scaffolds.

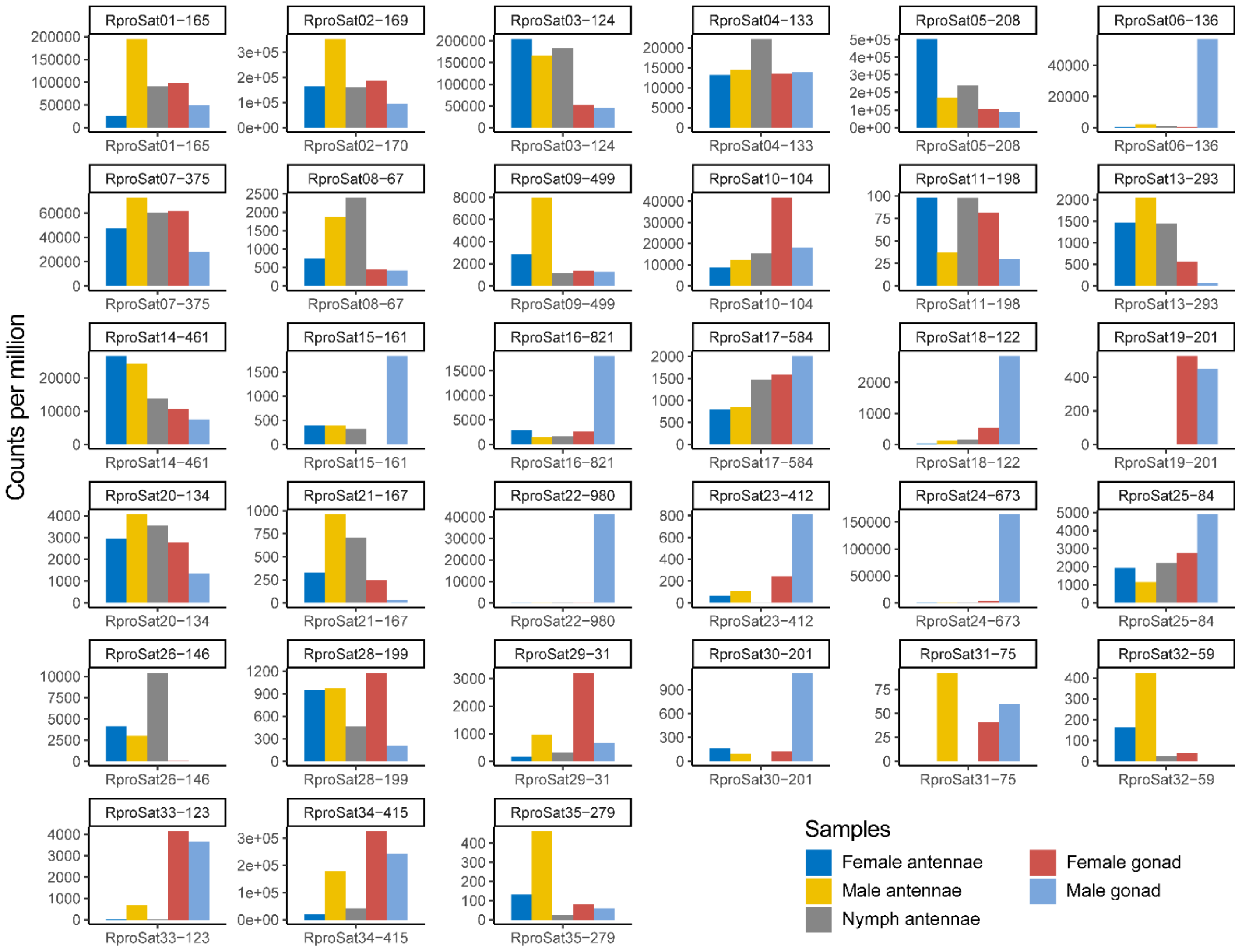

Figure S6. SatDNA transcription in different tissues.

Table S1: Estimations of genome abundance and nucleotide divergence of satDNA families and subfamilies found in *Rhodnius prolixus* genome sequenced in this work (Rpro) and in pseudo-reads generated from *Rhodnius prolixus* genome assembly (GCA_000181055.3) (RproSim). Subfamilies´ names are followed by the size of their consensus sequence. Table also shows the same data for available NCBI *Rhodnius prolixus* raw reads from genomic DNA: Rpro1 (SRR6749969), Rpro2 (SRR6749971), Rpro3 (SRR6749972) and Rpro4 (SRR6749978).

SatDNA Family	SatDNA Subfamily	Rpro Genome Prop. (%)	Rpro Kimura Diverg. (%)	RproSim Genome Prop. (%)	RproSim Kimura Diverg. (%)	Rpro1 Genome Prop. (%)	Rpro1 Kimura Diverg. (%)	Rpro2 Genome Prop. (%)	Rpro2 Kimura Diverg. (%)	Rpro3 Genome Prop. (%)	Rpro3 Kimura Diverg. (%)	Rpro4 Genome Prop. (%)	Rpro4 Kimura Diverg. (%)
RproSat01-165		2.131	13.42	1.159	16.10	2.723	12.87	3.075	12.73	2.652	12.97	2.504	12.48
	CL4-165	0.772	12.26	0.398	12.24	1.084	11.77	1.229	11.92	1.046	11.89	1.005	11.83
	CL11-166	0.577	17.31	0.335	17.39	0.650	16.33	0.687	16.3	0.570	16.24	0.590	16.16
	CL53-165	0.137	16.97	0.104	17.75	0.161	16.69	0.170	16.92	0.183	16.15	0.152	16.78
	CL64-166	0.093	6.42	0.058	6.35	0.108	5.99	0.135	6.17	0.115	6.16	0.105	6.22
	CL79A-178	0.074	17.98	0.032	18.08	0.100	16.32	0.112	16.44	0.102	16.13	0.088	15.84
	CL79B-166	0.060	21	0.029	21.73	0.078	19.32	0.092	19.34	0.084	19.73	0.074	18.78
	CL91-166	0.043	12.63	0.021	13.63	0.066	13.57	0.071	14.26	0.063	14.07	0.056	13.55
	CL82-178	0.035	14.24	0.006	19.37	0.037	13.23	0.050	11.04	0.041	11.86	0.033	11.85
	CL157-166	0.032	14.21	0.018	13.94	0.053	14.7	0.068	13.6	0.075	13.05	0.054	13.57
	CL193-165	0.025	23.15	0.019	22.55	0.027	20.66	0.028	23.22	0.033	20.21	0.031	20.56
	CL176-165	0.023	13.29	0.014	22.52	0.027	12.29	0.035	12.12	0.025	13.29	0.029	10.6
	CL162-165	0.023	12.64	0.013	13.9	0.024	11	0.037	11.09	0.030	11.53	0.028	10.75
	CL174-165	0.017	9.77	0.008	14.54	0.024	10.51	0.028	9.79	0.018	10.73	0.023	10.22
	CL341-165	0.017	22.23	0.010	25.77	0.017	19.84	0.015	19.32	0.015	20.28	0.014	20.17
	CL169-166	0.016	4.34	0.001	7	0.010	3.7	0.019	3.55	0.013	3.92	0.010	4.36
	CL345-165	0.015	17.92	0.010	21.82	0.020	16.43	0.022	15.09	0.018	15.98	0.019	14.93
	CL195-165	0.014	12.04	0.005	13.8	0.025	14.1	0.034	12.77	0.027	16.17	0.016	16.1
	CL256-167	0.013	20.41	0.006	25.4	0.017	17.74	0.021	17.24	0.016	18.67	0.014	17.93
	CL206-167	0.013	11.54	0.008	11	0.016	11.19	0.017	11.25	0.015	10.77	0.016	11.7
	CL217-167	0.012	5.14	0.002	12.57	0.012	6.82	0.007	5.69	0.006	7.6	0.009	5.38
	CL350-165	0.012	21.67	0.008	24.06	0.023	16.28	0.021	16.45	0.012	17.71	0.014	18.38
	CL227-168	0.010	8.62	0.003	18.36	0.011	17.67	0.015	14.23	0.017	13.04	0.012	13.55
	CL252-165	0.010	7.88	0.004	12.2	0.016	10.85	0.015	12.13	0.015	9.99	0.014	9.19
	CL232-165	0.009	9.91	0.006	9.98	0.015	9.43	0.020	9.03	0.011	9.92	0.013	8.64
	CL231-165	0.009	11.18	0.005	12.47	0.014	11.84	0.013	12.83	0.013	13.22	0.010	12.44
	CL300-165	0.008	15.14	0.004	14.1	0.012	15.42	0.010	16.34	0.012	16.52	0.010	15.64

	CL321-165	0.007	8.55	0.003	11.13	0.011	10.41	0.019	7.98	0.013	7.82	0.009	9.79
	CL292-165	0.007	9.15	0.003	16.23	0.010	7.51	0.013	5.97	0.008	9.86	0.014	5.75
	CL417-165	0.006	20.05	0.003	24.09	0.007	12.24	0.008	15.3	0.006	15.27	0.004	12.12
	CL334-165	0.006	18.45	0.004	18.75	0.005	12.89	0.006	15.17	0.006	11.22	0.005	10.79
	CL246-167	0.006	15.23	0.003	14.09	0.009	14.31	0.012	14.63	0.011	13.52	0.007	13.13
	CL354-173	0.006	10.97	0.003	17.35	0.005	11.69	0.005	10.04	0.006	9.49	0.005	8.92
	CL396-165	0.005	19.31	0.003	17.46	0.006	13.36	0.007	13.45	0.004	17.88	0.005	11.03
	CL335-165	0.005	6.65	0.001	15.46	0.008	9.63	0.011	10.05	0.006	12.64	0.004	13.39
	CL266-178	0.005	12.14	0.001	13.02	0.003	9.77	0.006	11.03	0.002	9.4	0.004	8.47
	CL336-154	0.004	8.14	0.003	16.83	0.004	8.89	0.005	8.2	0.003	8.74	0.003	10.49
	CL401-165	0.003	15.97	0.001	17.23	0.007	17.36	0.008	14.35	0.008	15.05	0.004	18.06
	CL356-165	0.003	5.52	0.0005	7.47	0.003	7.34	0.003	9.27	0.004	7.08	0.003	7.26
RproSat02-169		1.936	9.81	0.883	11.35	2.318	8.42	2.329	8.91	2.370	8.72	2.100	8.64
	CL2-169	1.147	5.2	0.586	5.14	1.331	4.89	1.353	4.98	1.354	4.89	1.204	4.92
	CL6-170	0.729	5.93	0.266	6.03	0.891	5.65	0.867	5.73	0.916	5.59	0.815	5.7
	CL106-169	0.036	14.63	0.019	13.66	0.058	12.99	0.068	13.64	0.064	13.1	0.051	12.35
	CL239-170	0.018	16.06	0.010	18.08	0.031	11.29	0.034	11.05	0.029	12.95	0.024	11.85
	CL323-170	0.006	7.22	0.001	13.86	0.008	7.28	0.007	9.15	0.007	7.08	0.005	8.39
RproSat03-124		1.182	8.81	1.35	8.94	1.476	7.81	1.397	7.85	1.375	7.93	1.326	7.72
	CL1-124	0.671	7.27	0.725	7.34	0.908	6.86	0.872	6.85	0.848	6.92	0.857	6.93
	CL46-130	0.510	10.35	0.627	10.54	0.567	8.75	0.525	8.84	0.527	8.93	0.469	8.5
RproSat04-133		0.861	17.38	0.510	21.78	0.979	16.10	0.967	17.19	1.023	15.79	0.940	15.63
	CL36-133	0.328	19.69	0.200	19.77	0.403	19.28	0.373	19.43	0.404	19.38	0.358	19.44
	CL88-134	0.100	21.17	0.016	18.6	0.092	17.81	0.086	17.39	0.081	17.63	0.073	17.43
	CL85-133	0.079	18.03	0.066	17.75	0.089	15.88	0.089	16.53	0.093	16	0.077	16.46
	CL353-133	0.046	23.72	0.041	23.08	0.037	21.56	0.042	21.28	0.044	21.1	0.040	21.44
	CL395-132	0.045	26.54	0.033	26.96	0.044	24.12	0.048	19.66	0.050	22.36	0.040	23.36
	CL131-132	0.044	19.31	0.042	17.88	0.065	16.74	0.076	17.02	0.075	16.99	0.069	17.5
	CL183-133	0.027	18	0.013	29.04	0.025	14.05	0.027	11.81	0.029	10.59	0.038	8.58
	CL80-134	0.026	23.85	0.013	26.6	0.024	22.22	0.018	22.88	0.028	21.23	0.023	21.12
	CL386-133	0.017	25.64	0.007	28.38	0.015	24.17	0.015	20.82	0.017	22.61	0.017	22.44
	CL244-131	0.017	20.18	0.011	20.99	0.017	18.68	0.019	18.34	0.019	19	0.022	17.79
	CL148-133	0.016	11.51	0.007	17.4	0.028	9.16	0.039	9.47	0.034	7.99	0.028	10.25
	CL154-133	0.015	16.1	0.009	18.15	0.010	18.3	0.012	18.23	0.014	17.57	0.015	14.83
	CL199-132	0.014	15.73	0.004	19.99	0.016	16.14	0.017	14.85	0.013	15.54	0.017	15.57
	CL219-132	0.012	9.49	0.007	22.87	0.018	9.28	0.029	7.82	0.024	8.56	0.021	7.86
	CL212-133	0.011	10.5	0.006	13.49	0.010	11.78	0.012	9.07	0.013	10.03	0.010	10.24
	CL310-134	0.011	16.84	0.005	35.05	0.026	8.55	0.011	17.4	0.028	7.85	0.041	7.34
	CL366-133	0.011	11.85	0.004	20.27	0.010	18.37	0.014	17.29	0.009	18.31	0.009	18.8

	CL288-132	0.008	20.12	0.004	23.35	0.010	16.92	0.008	18.47	0.010	16.64	0.007	18.95
	CL267-132	0.007	11.02	0.006	10.16	0.011	9.21	0.013	10.29	0.009	9.33	0.011	10.25
	CL423-132	0.007	14.8	0.004	32.56	0.009	22.25	0.005	28.34	0.008	21.42	0.009	20.84
	CL420-132	0.007	15.24	0.004	21.37	0.007	13.78	0.003	24.28	0.010	19.59	0.004	16.07
	CL355-132	0.006	14.06	0.003	14.59	0.004	10.09	0.006	11.73	0.006	11.41	0.005	8.61
	CL331-133	0.004	16.32	0.004	22.67	0.008	11.96	0.005	22.98	0.007	12	0.006	14.41
RproSat05-208		0.460	15.48	0.840	13.38	0.701	12.09	0.654	12.07	0.664	12.19	0.634	12.11
RproSat06-136		0.320	12.42	0.133	12.29	0.176	11.32	0.011	10	0.041	10.98	0.154	11.42
RproSat07-375		0.200	7.77	0.101	9.13	0.193	6.35	0.187	6.94	0.186	6.56	0.163	6.75
RproSat08-67		0.194	20.58	0.173	20.64	0.261	18.46	0.245	18.37	0.262	18.18	0.245	18.56
RproSat09-499		0.107	12.2	0.046	11.91	0.097	12.09	0.096	12.15	0.089	12.37	0.090	12.17
RproSat10-104		0.085	10.1	0.099	10.19	0.132	9.52	0.137	9.75	0.129	9.75	0.131	9.51
RproSat11-198		0.073	2.23	0.008	3.81	0.100	2.41	0.125	3.33	0.063	2.13	0.125	1.53
RproSat12-41		0.069	10.03										
RproSat13-293		0.067	28.28	0.078	28.74	0.044	24.82	0.040	25.73	0.043	25.57	0.038	24.9
RproSat14-461		0.062	6.19	0.015	8.2	0.061	4.83	0.040	5.58	0.058	4.74	0.049	4.88
RproSat15-161		0.052	8.38	0.013	7.8	0.031	7.69	0.001	7.31	0.009	6.67	0.029	6.18
RproSat16-821		0.044	6.83	0.025	14.24	0.065	6.05	0.063	5.53	0.056	5.21	0.053	6.36
RproSat17-584		0.027	11.71	0.016	22.49	0.025	8.9	0.028	6.8	0.026	7.76	0.025	7.54
RproSat18-122		0.019	25.27	0.026	24.56	0.028	22.77	0.026	22.32	0.027	22.19	0.025	23.37
RproSat19-201		0.019	19.52	0.020	23.86	0.014	15.94	0.020	13.06	0.015	15.91	0.014	17.32
RproSat20-134		0.017	16.23	0.018	16.96	0.018	12.52	0.017	13.99	0.018	13.75	0.020	12.46
RproSat21-167		0.016	16.25	0.013	14.21	0.020	13.78	0.024	13.71	0.022	13.12	0.018	14.28
RproSat22-980		0.013	4.75	0.005	6.33	0.012	4.29	0.012	4.48	0.010	5.01	0.011	3.45
	CL166A-980	0.012	5.57	0.004	5.41	0.011	4.68	0.011	4.23	0.010	4.24	0.010	4.13
	CL166B-745	0.002	3.92	0.001	7.25	0.001	3.89	0.002	4.73	0.001	5.78	0.001	2.76
RproSat23-412		0.010	5.59	0.004	5.77	0.012	4.73	0.005	6.31	0.004	5.19	0.009	5.31
RproSat24-673		0.009	2.89	0.002	5.43	0.013	2.96	0.011	3.25	0.012	2.8	0.013	2.72
RproSat25-84		0.009	25.75	0.008	26.89	0.010	24.62	0.009	23.75	0.009	24.41	0.008	24.19
RproSat26-146		0.009	3.55	0.002	10.62	0.005	4.28	0.004	4.42	0.011	3.23	0.005	3.8
RproSat27-187		0.008	13.06										
RproSat28-199		0.008	12.72	0.010	12.02	0.011	9.4	0.014	8.3	0.011	8.51	0.009	9.05
RproSat29-31		0.006	11.21	0.013	10.75	0.010	10.22	0.013	10.9	0.008	10.61	0.010	9.68
RproSat30-201		0.006	3.02	0.002	7.77	0.009	1.82	0.002	3.52	0.006	2.11	0.005	5.51
RproSat31-75		0.005	2.64	0.0002	2.37	0.005	1.97	0.007	2.06	0.005	2.04	0.005	1.72
RproSat32-59		0.004	0.88	0.0003	0.22	0.009	0.52			0.005	0.74	0.003	0.5
RproSat33-123		0.003	0.96			0.003	0.45	0.003	0.76	0.002	0.51	0.002	0.39
RproSat34-415		0.003	5.15	0.003	7.15	0.003	10.26	0.003	6.33	0.005	6.62	0.003	7.35
RproSat35-279		0.003	5.71	0.001	7.02	0.002	8.16	0.003	3.1	0.004	5.69	0.003	6.08

RproSat36-40	0.003	4.17	0.001	4.76	0.001	4.8	0.000	3	0.002	4.6	0.001	4.43
RproSat37-98	0.0005	6.96			0.001	10.03	0.0002	9.18	0.0003	2.89	0.0002	2.48
Telomeric repeat	0.003	13.86	0.007	11.91	0.048	2.38	0.035	2.75	0.030	3.06	0.037	2.47
(GATA)n repeat	0.001	10.09	0.002	12.21	0.005	2.27	0.002	8.36	0.002	7.7	0.004	4.21
**Total**	**8.046**		**5.585**		**9.622**		**9.608**		**9.255**		**8.809**	
Mean		10.41		12.17		9.00		9.14		8.77		8.70
SD		6.88		7.08		6.38		6.16		6.38		6.49
Median		9.92		11.05		8.29		7.85		7.19		7.05

Table S2: SatDNA transcription on different tissues. Combined column refers to sum of all samples transcription for each satDNA family.

	Samples					
**SatDNA Family**	**Female antennae**	**Male antennae**	**Nymph antennae**	**Female gonad**	**Male gonad**	**Combined**
RproSat01-165	25875.90	194766.25	90631.36	98288.63	49236.27	458798.40
RproSat02-169	164382.27	352430.66	161782.36	188298.61	95682.18	962576.08
RproSat03-124	203899.45	166091.61	183193.65	52106.77	45935.85	651227.33
RproSat04-133	13183.29	14567.82	22169.00	13432.51	13891.77	77244.39
RproSat05-208	502077.44	170443.52	240119.34	105958.55	88481.26	1107080.11
RproSat06-136	228.99	2194.39	782.15	284.07	56887.24	60376.84
RproSat07-375	47433.69	72728.47	60347.58	61440.54	28023.57	269973.85
RproSat08-67	752.40	1880.91	2395.33	446.40	420.05	5895.08
RproSat09-499	2878.73	7966.20	1148.78	1379.77	1260.16	14633.65
RproSat10-104	8636.20	12336.55	15251.88	41677.30	18062.30	95964.23
RproSat11-198	98.14	36.88	97.77	81.16	30.00	343.95
RproSat13-293	1472.08	2046.87	1442.08	568.14	60.01	5589.19
RproSat14-461	26595.58	24359.61	13858.68	10713.54	7560.96	83088.37
RproSat15-161	392.55	387.25	317.75	0.00	1830.23	2927.78
RproSat16-821	2846.02	1438.34	1613.18	2597.22	18002.29	26497.06
RproSat17-584	785.11	848.25	1466.53	1582.68	2010.26	6692.83
RproSat18-122	32.71	129.08	146.65	527.56	2850.36	3686.37
RproSat19-201	0.00	0.00	0.00	527.56	450.06	977.62
RproSat20-134	2944.16	4056.86	3544.11	2759.55	1350.17	14654.85
RproSat21-167	327.13	958.89	708.82	243.49	30.00	2268.34
RproSat22-980	0.00	36.88	97.77	40.58	41165.24	41340.47
RproSat23-412	65.43	110.64	0.00	243.49	810.10	1229.66
RproSat24-673	457.98	295.04	1075.45	4017.58	164030.87	169876.93
RproSat25-84	1930.06	1143.30	2199.79	2759.55	4890.62	12923.32
RproSat26-146	4154.54	2987.33	10412.34	40.58	30.00	17624.79
RproSat28-199	948.67	977.33	464.40	1176.87	210.03	3777.30
RproSat29-31	163.56	977.33	317.75	3205.95	660.08	5324.68
RproSat30-201	163.56	92.20	0.00	121.74	1110.14	1487.65
RproSat31-75	0.00	92.20	0.00	40.58	60.01	192.79
RproSat32-59	163.56	424.13	24.44	40.58	0.00	652.71
RproSat33-123	32.71	682.29	24.44	4139.32	3660.47	8539.23
RproSat34-415	19202.47	177856.51	42309.30	324815.10	242700.88	806884.27
RproSat35-279	130.85	461.01	24.44	81.16	60.01	757.47

Table S3: Results of Spearman correlation between satDNA transcription and abundance in different samples.

Samples	r_s	p-value
Combined	0.52	0.001728
Female antennae	0.58	0.0004624
Male antennae	0.55	0.0008323
Nymph antennae	0.64	6.42E-05
Female gonad	0.40	0.0197
Male gonad	0.41	0.01738

Table S4. Designed oligonucleotides for three main satellite DNA families of *Rhodnius prolixus*.

SatDNA family	Oligonucleotide	Sequence
RproSat01-165	Rpro-165-F	5′ CTCTAAATATCTCGTTTCCTACTCGACAG
	Rpro-165-R	5′ CTTTAACTTCAAATATCTCGAAAACTACTCG
RproSat02-169	Rpro-169-F	5′ ATTATGGCCGTTCTATATCTCCCAC
	Rpro-169-R1	5′ ATCTTCTACATTTATAGATCTGTGCAC
	Rpro-169-R2	5′ GTACAAGAGAACAAAATTCCATACCA
RproSat03-124	Rpro-124-F	5′ CGGGTCTAGCCTATAACCGC
	Rpro-124-R	5′ TAGACCCGCAAAGCTAAGATGTATTG
